# Changes of corneal topographic measurements and higher-order aberrations after surgery for exotropia

**DOI:** 10.1371/journal.pone.0202864

**Published:** 2018-08-24

**Authors:** Seok Hyun Bae, Dong Gyu Choi

**Affiliations:** 1 Department of Ophthalmology, Central Physical Examination Office, Daegu, Korea; 2 Department of Ophthalmology, Kangnam Sacred Heart Hospital, Hallym University College of Medicine, Seoul, Korea; Faculty of Medicine, Cairo University, EGYPT

## Abstract

**Purpose:**

To analyze changes in corneal topographic measurements and higher-order aberrations (HOAs) after horizontal muscle surgery for exotropia.

**Design:**

Retrospective, observational study.

**Methods:**

A total of 131 eyes of 121 patients who had undergone surgery for exotropia were included. The eyes with unilateral lateral rectus (ULR) or bilateral lateral rectus (BLR) recession(s) were assigned to group A, and those with unilateral lateral rectus recession & medial rectus resection (R&R) to group B. Corneal topographic measurements and HOAs were compared between the preoperative and postoperative periods using a Placido-dual Scheimpflug analyzer (Galilei 2^TM^, Ziemer, Port., Switzerland) for each group.

**Results:**

In group A, simulated keratometery (sim K) was significantly changed until 3 months postoperatively relative to the preoperative value (postoperative 1 week, p = 0.017; 1 month, p = 0.037; and 3 months, p = 0.023, respectively). All steep K (steep sim K, steep-Kpost, and TCP-steep K) parameters also were significantly changed at postoperative 1 month (p<0.001, p = 0.015, p<0.001, respectively), but not at 3 months. Among the higher-order aberrations, spherical aberration (Z_4_^0^) and secondary horizontal astigmatism (Z_4_^2^) at postoperative 1 week had significantly changed from the preoperative values, as had horizontal quadrafoil (Z_4_^4^) at 1 month. However, in group B, only vertical quadrafoil (Z_4_^-4^) showed statistically significant changes, at postoperative 1 and 3 months. None of the other postoperative parameters was significantly different from the corresponding preoperative value.

**Conclusion:**

Lateral rectus recession induced changes in both corneal topographic measurements and HOAs, whereas R&R did so only in HOAs. These changes might explain some patients' complaints about poor quality of vision.

## Introduction

Surgical correction of exotropia, to restore the normal ocular position by weakening the lateral rectus muscle with/without strengthening the medial rectus muscle through changing the orientation of their action plan, has been the main treatment for deviations [1]. Many studies have shown that changes of refractive error or astigmatic power are transient and not clinically important in most cases [2–4]; meanwhile, it has been reported that such changes remain stable long term in some patients [5–7]. Also, there have been many studies on topographic changes following strabismus surgery. Recession of a single rectus muscle typically effects a decrease of corneal curvature in the meridian of the recessed muscle, though paradoxically, it occasionally increases the focusing power along this meridian [4, 6–10]. Incidence of increased with-the-rule astigmatism after horizontal muscle recess-resect procedures also has been reported [11].

Recently, many imaging techniques for anterior corneal assessment have been developed. Orbscan^TM^ (Bausch & Lomb, Rochester, NY), which uses Placido disk technology to display conventional corneal topography, was the first commercially viable hybrid system [12]. A hybrid Placido-Scheimpflug device, Pentacam^TM^ (Oculus, Inc., Wetzlar, Germany), became commercially available in 2002 [13]. Lately, the Placido-dual Scheimpflug analyzer (Galilei^TM^, Ziemer, Port., Switzerland), which uses two Scheimpflug cameras, has been introduced as well [14]. The dual-camera system derives images from both sides, which minimizes the effect of decentration due to eye movements on corneal pachymetry and posterior corneal curvature measurements [15].

In normal eyes, most of the optical irregularity is caused by second-order aberrations, which are called refractive errors. Higher-order aberrations (HOAs), by contrast, are a relatively small component of optical irregularity. However, many authors believe that HOAs might play a significant role in reducing retinal image quality [16, 17]. HOAs, unlike lower-order aberrations such as myopia, hyperopia, and astigmatism, cannot be corrected with spectacles. HOAs usually have been studied for the purposes of refractive surgery [18, 19].

Curiously, patients who have undergone strabismus surgery sometimes complain about their quality of vision even though the visual acuity or refractive error was not changed. We hypothesized that such patients might be influenced by postoperative changes of HOAs, as HOAs have been known to affect vision quality. However, there remains only scant information in the literature on post-strabismus-surgery changes in corneal topographic measurements or HOAs. Therefore, the aim of this study was to analyze surgically induced changes of corneal topographic measurements and HOAs using Galilei after surgery for exotropia.

## Materials and methods

### Study population

In this retrospective study, the medical records of 121 patients who had undergone surgery for exotropia between August 2012 and February 2017 with a postoperative follow-up period of 3 months or more were reviewed. Patients were excluded if they had a history of any eye surgery including strabismus surgery and corneal surgery, ocular disease, or neurologic disorder such as Down syndrome or cerebral palsy. Data from any patient who did not cooperate with the testing were excluded. The study was performed in accordance with the tenets of the Declaration of Helsinki, and was reviewed and approved by the Institutional Review Board/Ethics Committee of Hallym University Medical Center with an understanding on exemption from the informed consent for the study of retrospective collection of the clinical data. All data were anonymized before we accessed them.

### Ophthalmologic evaluation

All of the patients underwent a complete ophthalmologic examination prior to their exotropia surgery. We noted preoperative characteristics including age at surgery, sex, mean angle of exodeviation at distance and near, best-corrected visual acuity (BCVA), refractive error, and the slit-lamp examination result. Deviation angles were measured by the alternate prism cover test for distance (6 m) and near (33 cm) in all 9 positions of gaze using accommodative targets and the patients’ best optical correction. Refractive error was measured by streak retinoscopy after cycloplegia using 1% cyclopentolate hydrochloride (Cyclogyl®, Alcon Lab. Inc., Fort Worth, TX, USA) and 1% tropicamide (Mydriacyl®, Alcon Lab. Inc.). Amblyopia was defined as a between-eye difference of 2 lines or more in visual acuity.

### Measurement of corneal topographic measurements and HOAs

We measured the corneal topography and HOAs using the Placido-dual Scheimpflug analyzer (Galilei 2, Ziemer, Port., Switzerland). All of the measurements were performed by three experienced examiners, one examiner having been assigned randomly for each case. The patients were instructed to blink completely just before each measurement.

The corneal topographic measurements determined from the Scheimpflug images were as follows: (1) mean simulated keratometry (sim K), corneal dioptric power in the flattest meridian (flat sim K) and steepest meridian (steep sim K) of the 3.0 mm central zone; (2) posterior curvature-average (mean-Kpost), posterior curvature-flat K (flat Kpost), posterior curvature-steep K (steep Kpost); (3) total corneal power (TCP)-average K (TCP-mean K), TCP-flat K, and TCP-steep K.

The HOAs were shown in the wavefront report of Galilei. The wavefront aberration data were analysed with a 6 mm pupil size. The elevation data from the Scheimpflug images were combined to form a 3-dimensional reconstruction of the corneal structure. Internal software automatically converted the corneal elevation profile to corneal wavefront data using Zernicke polynomials. The data analysis included the third-order Zernike components (Z_3_^-3^ to Z_3_^3^) and fourth-order Zernike components (Z_4_^−4^ to Z_4_^4^).

### Surgery

All of the patients underwent exotropia surgery under general anesthesia by one surgeon (D.G.C.). Unilateral lateral rectus (ULR) recession, bilateral lateral rectus (BLR) recession, or unilateral lateral rectus recession & medial rectus resection (R&R) was performed. The surgical method was selected by the surgeon, who had no preference for BLR recession or R&R. When we performed lateral rectus recession (ULR or BLR), we performed the recession procedure using the scleral suture method, not the hang-back technique. Lateral rectus (LR) recession was performed through a temporal limbal conjunctival incision. After the muscle tendon was dissected from the sclera using curved Stevens tenotomy scissors, the needles were inserted through the sclera, entering the tissue at the mark and emerging slightly lateral and parallel to the limbus. The surgical dosages of each group were based on the largest deviation angles at distance deviation, as indicated in Table 1. Some cases with exotropia of < 25 prism diopters (PD) both at distant and near fixation underwent ULR recession.

**Table 1:** Surgical dosages for exodeviation.

PD	BLR recession (mm)	R&R (mm)	ULR recession (mm)
**15**	4.0	4.0/3.0	8.0
**20**	5.0	5.0/4.0	9.0
**25**	6.0	6.0/5.0	10.0
**30**	7.0	7.0/5.5	
**35**	7.5	7.5/6.0	
**40**	8.0	8.0/6.5	
**45**	8.5	8.5/7.0	
**50**	9.0	9.0/7.0	

PD = Prism diopters; BLR = Bilateral lateral rectus; R&R = Unilateral lateral rectus recession & medial rectus resection; ULR = Unilateral lateral rectus

### Grouping

The eyes with lateral rectus recession in patients who had undergone ULR or BLR recession(s) were assigned to group A, and those with R&R were assigned to group B.

### Main outcome measures

The main outcome measures were the postoperative changes of corneal topographic measurements and of HOA in each group. Additionally, the correlation between the recession amount and the postoperative change of HOAs (which showed significant postoperative changes in group A) was investigated.

### Statistical analysis

Data were analyzed using SPSS software version 24 (SPSS Inc., Chicago, IL). Continuous variables were expressed as mean ± standard deviations. The Pearson chi-square test and Mann-Whitney U-test were used to compare the preoperative characteristics between the groups. The mixed model was used to compare the pre- and postoperative corneal topographic measurements and HOAs in each group. The correlation between the recession amount and the postoperative change of HOAs was evaluated by Pearson correlation analysis. P values less than 0.05 were considered statistically significant.

## Results

Table 2 shows the preoperative demographic data on each group. The mean preoperative exodeviations were 20.9 ± 5.6 PD at distance and 21.5 ± 5.3 PD at near in group A, and 29.1 ± 10.0 PD and 31.3 ± 10.1 PD in group B (p <0.001 at both distance and near, Mann-Whitney U-test). The reason for the significantly smaller preoperative angles in Group A is that group A included unilateral lateral muscle recession (one-muscle surgery) for small-angle exotropia as well as bilateral lateral rectus recession (two-muscle surgery), while group B included only the recess-resect procedure (two-muscle surgery).

**Table 2:** Preoperative demographic data.

Variables	Group A (53 patients, 63 eyes)	Group B (68 patients, 68 eyes)	p-value
Sex (Male/Female)	25/28	34/34	0.757*
Age at surgery (years)	9.1 ± 5.9 (2–41)	12.9 ± 10.4 (3–50)	0.103†
Preoperative angle of exodeviation (PD)			
at distance	20.9 ± 5.6	29.1 ± 10.0	<0.001†
at near	21.5 ± 5.3	31.3 ± 10.1	<0.001†
BCVA (logMAR)			
Dominant eye	0.01 ± 0.03	0.03 ± 0.07	0.474†
Non-dominant eye	0.04 ± 0.14	0.10 ± 0.29	0.659†
Amblyopia (n, %)	2/53 (3.8%)	5/68 (7.4%)	0.462‡
Refractive error (D)			
Spherical	-1.14 ± 2.09	-1.42 ± 3.85	0.944†
Cylinder	-0.89 ± 0.72	-1.0 ± 0.93	0.307†

Group A = Eyes with lateral rectus recession in patients who had undergone unilateral or bilateral lateral rectus recession(s)

Group B = Eyes with lateral rectus recession & medial rectus resection

PD = prism diopters, BCVA = best-corrected visual acuity, D = diopters

*Pearson chi-square test

†Mann-Whitney U-test

‡Fisher's Exact Test

### Postoperative changes of corneal topographic measurement

Table 3 shows the results of corneal topographic measurements in group A before and after surgery. The parameter sim K had significantly changed by postoperative 1 week (p = 0.017, mixed model), 1 month (p = 0.037) and 3 months (p = 0.023) compared with the preoperative value. TCP-steep K had significantly changed at postoperative 1 week (p = 0.017). All steep K (steep sim K, steep-Kpost, TCP-steep K) had changed at postoperative 1 month (p<0.001, p = 0.015, p<0.001, respectively), but had reverted to unchanged status at 3 months.

**Table 3:** Comparison of preoperative and postoperative data on corneal structure in group A.

Parameters	Preoperative	Postoperative 1 week		Postoperative 1 month		Postoperative 3 months	
		value	p-value*	value	p-value*	value	p-value*
Sim K (D)	42.18 ± 1.44	**42.92 ±1.70**	**0.017**	**43.69 ±5.11**	**0.037**	**42.71 ±1.61**	**0.023**
Flat sim K (D)	42.18 ± 1.44	42.04 ±1.82	0.574	42.10 ±1.82	0.143	41.77 ±1.47	0.532
Steep sim K (D)	43.67 ± 1.81	43.79 ±1.79	0.082	**43.92 ±1.89**	**<0.001**	43.65 ±1.86	0.119
Mean-Kpost (D)	-6.17 ± 0.30	-6.14 ±0.48	0.375	-6.22 ±0.57	0.696	-6.13 ±0.28	0.880
Flat-Kpost (D)	-5.96 ± 0.32	-5.87 ±0.57	0.203	-5.89 ±0.71	0.301	-5.91 ±0.28	0.500
Steep-Kpost (D)	-6.37 ± 0.39	-6.40 ±0.55	0.949	**-6.45 ±1.32**	**0.015**	-6.36 ±0.41	0.983
TCP-mean K (D)	41.91 ± 1.61	41.99 ±1.76	0.428	41.94 ±1.79	0.196	41.77 ±1.59	0.221
TCP-flat K (D)	41.19 ± 1.52	41.10 ±1.89	0.418	41.02 ±2.08	0.290	40.84 ±1.44	0.628
TCP-steep K (D)	42.66 ± 1.83	**42.77 ±1.77**	**0.017**	**42.82 ±1.68**	**<0.001**	42.95 ±2.52	0.217

Group A = Eyes with lateral rectus recession in patients who had undergone unilateral or bilateral lateral rectus recession(s)

Sim K = simulated keratometry; Kpost = posterior curvature keratometry; TCP = total corneal power; K = keratometry

* Mixed model for comparison between preoperative and postoperative variables

However, for each parameter in group B, there was no significant pre-to-postoperative difference in the mean variance (all p>0.05) (Table 4).

**Table 4:** Comparison of preoperative and postoperative data on corneal structure in group B.

Parameters	Preoperative	Postoperative 1 week		Postoperative 1 month		Postoperative 3 months	
		value	p-value*	value	p-value*	value	p-value*
Sim K (D)	43.09 ± 1.47	43.24 ±1.42	0.987	43.22 ±1.46	0.141	43.09 ±1.19	0.849
Flat sim K (D)	42.27 ± 1.44	42.36 ±1.37	0.379	42.00 ±2.13	0.269	42.26 ±1.23	0.628
Steep sim K (D)	43.91 ± 1.70	44.11 ±1.69	0.298	44.44 ±1.88	0.645	43.91 ±1.32	0.307
Mean-Kpost (D)	-6.23 ±0.44	-6.16 ±0.60	0.786	-6.22 ±0.56	0.551	-6.24 ±0.36	0.908
Flat-Kpost (D)	-5.92 ± 0.66	-5.83 ±0.85	0.297	-6.02 ±0.48	0.329	-5.98 ±0.23	0.796
Steep-Kpost (D)	-6.49 ± 0.43	-6.50 ±0.55	0.750	-6.07 ±1.78	0.637	-6.51 ±0.59	0.991
TCP-mean K (D)	42.04 ±1.54	42.10 ±1.82	0.560	42.16 ±1.56	0.510	42.01 ±1.21	0.551
TCP-flat K (D)	41.22 ±1.54	41.37 ±1.42	0.751	40.96 ±2.42	0.209	41.24 ±1.25	0.894
TCP-steep K (D)	42.86 ±1.84	43.13 ±1.74	0.494	43.37 ±1.90	0.961	42.80 ±1.30	0.081

Group B = Eyes with lateral rectus recession & medial rectus resection

Sim K = simulated keratometry; Kpost = posterior curvature keratometry; TCP = total corneal power; K = keratometry

* Mixed model for comparison between preoperative and postoperative variables

### Postoperative changes of HOA

Tables 5 and 6 show the corneal HOAs with a 6 mm diameter central corneal zone preoperatively and at postoperative 1 week, 1 and 3 months in groups A and group B, respectively. The changes of third-order aberrations—vertical and horizontal corneal coma (Z_3_^-1^, Z_3_^1^) and trefoil (Z_3_^−3^, Z_3_^3^)—throughout the 3-month follow-up period after strabismus surgery were not significantly different from the preoperative value in both groups (all p>0.05).

**Table 5:** Preoperative and postoperative 1 week, 1 and 3 months corneal HOAs with 6 mm diameter central corneal zone in group A (mean ± SD, μm).

Type of aberration (Zernike Term)	Preoperative	Postoperative 1 week		Postoperative 1 month		Postoperative 3 months	
		value	p-value*	value	p-value*	value	p-value*
Third-order aberration							
Vertical trefoil (Z_3_^−3^)	-0.02 ± 0.30	0.01 ±0.43	0.365	0.00 ±0.17	0.085	-0.02 ±0.24	0.669
Vertical coma (Z_3_^−1^)	0.04 ± 0.33	0.00 ±0.33	0.117	0.06 ±0.17	0.098	0.06 ±0.21	0.649
Horizontal coma (Z_3_^1^)	-0.06 ± 0.29	-0.12 ±0.28	0.497	-0.07 ±0.29	0.436	0.01 ±0.26	0.341
Horizontal trefoil (Z_3_^3^)	-0.08 ± 0.18	-0.05 ±0.18	0.848	-0.07 ±0.19	0.759	-0.06 ±0.20	0.708
Fourth-order aberration							
Vertical quadrafoil (Z_4_^-4^)	0.03 ±0.09	0.02 ±0.15	0.598	-0.05 ±0.12	0.736	-0.08 ±0.19	0.750
Secondary vertical astigmatism (Z_4_^-2^)	0.00 ±0.06	0.01 ±0.07	0.650	-0.01 ±0.07	0.887	0.00 ±0.08	0.775
Spherical aberration (Z_4_^0^)	0.11 ±0.13	**0.10 ±0.11**	**0.002**	0.17 ±0.14	0.208	0.12 ±0.06	0.688
Secondary horizontal astigmatism (Z_4_^2^)	0.06 ±0.12	**0.08 ±0.13**	**0.018**	0.01 ±0.11	0.863	0.03 ±0.05	0.468
Horizontal quadrafoil (Z_4_^4^)	-0.08 ±0.14	-0.08 ±0.23	0.483	**-0.01 ±0.12**	**0.049**	-0.03 ±0.19	0.839

Group A = Eyes with lateral rectus recession in patients who had undergone unilateral or bilateral lateral rectus recession(s)

* Mixed model for comparison between preoperative and postoperative variables

**Table 6:** Preoperative and postoperative 1 week, 1 and 3 months corneal HOAs with 6 mm diameter central corneal zone in group B (mean ± SD, μm).

Type of aberration (Zernike Term)	Preoperative	Postoperative 1 week		Postoperative 1 month		Postoperative 3 months	
		value	p-value*	value	p-value*	value	p-value*
Third-order aberration							
Vertical trefoil (Z_3_^−3^)	-0.03 ±0.32	-0.12 ±0.54	0.657	0.12 ±0.36	0.918	-0.01 ±0.23	0.973
Vertical coma (Z_3_^−1^)	0.06 ±0.31	0.20 ±0.53	0.461	-0.07 ±0.36	0.956	0.02 ±0.25	0.972
Horizontal coma (Z_3_^1^)	0.03 ±0.28	-0.02 ±0.30	0.246	-0.02 ±0.28	0.688	0.02 ±0.22	0.629
Horizontal trefoil (Z_3_^3^)	-0.07 ±0.26	-0.10 ±0.38	0.476	-0.05 ±0.45	0.839	-0.06 ±0.20	0.692
Fourth-order aberration							
Vertical quadrafoil (Z_4_^-4^)	0.00 ±0.09	0.03 ±0.34	0.653	**-0.02 ±0.15**	**0.023**	**0.03 ±0.10**	**0.010**
Secondary vertical astigmatism (Z_4_^-2^)	0.02 ±0.08	0.03 ±0.10	0.828	0.02 ±0.09	0.212	0.03 ±0.70	0.712
Spherical aberration (Z_4_^0^)	0.15 ±0.08	0.07 ±0.19	0.497	0.11 ±0.13	0.119	0.13 ±.15	0.728
Secondary horizontal astigmatism (Z_4_^2^)	0.04 ±0.10	0.11 ±0.16	0.528	0.05 ±0.17	0.056	0.02 ±0.16	0.565
Horizontal quadrafoil (Z_4_^4^)	-0.07 ±0.16	-0.10 ±0.16	0.856	-0.16 ±0.25	0.172	-0.08 ±0.22	0.310

Group B = Eyes with lateral rectus recession & medial rectus resection

* Mixed model for comparison between preoperative and postoperative variables

In group A, the spherical aberration (Z_4_^0^) and secondary horizontal astigmatism (Z_4_^2^) significantly differed in their preoperative and postoperative 1 week values (p = 0.002, p = 0.018, respectively). Also in group A, horizontal quadrafoil (Z_4_^4^) showed a significant change at 1 month (p = 0.049).

**Fig 1:**
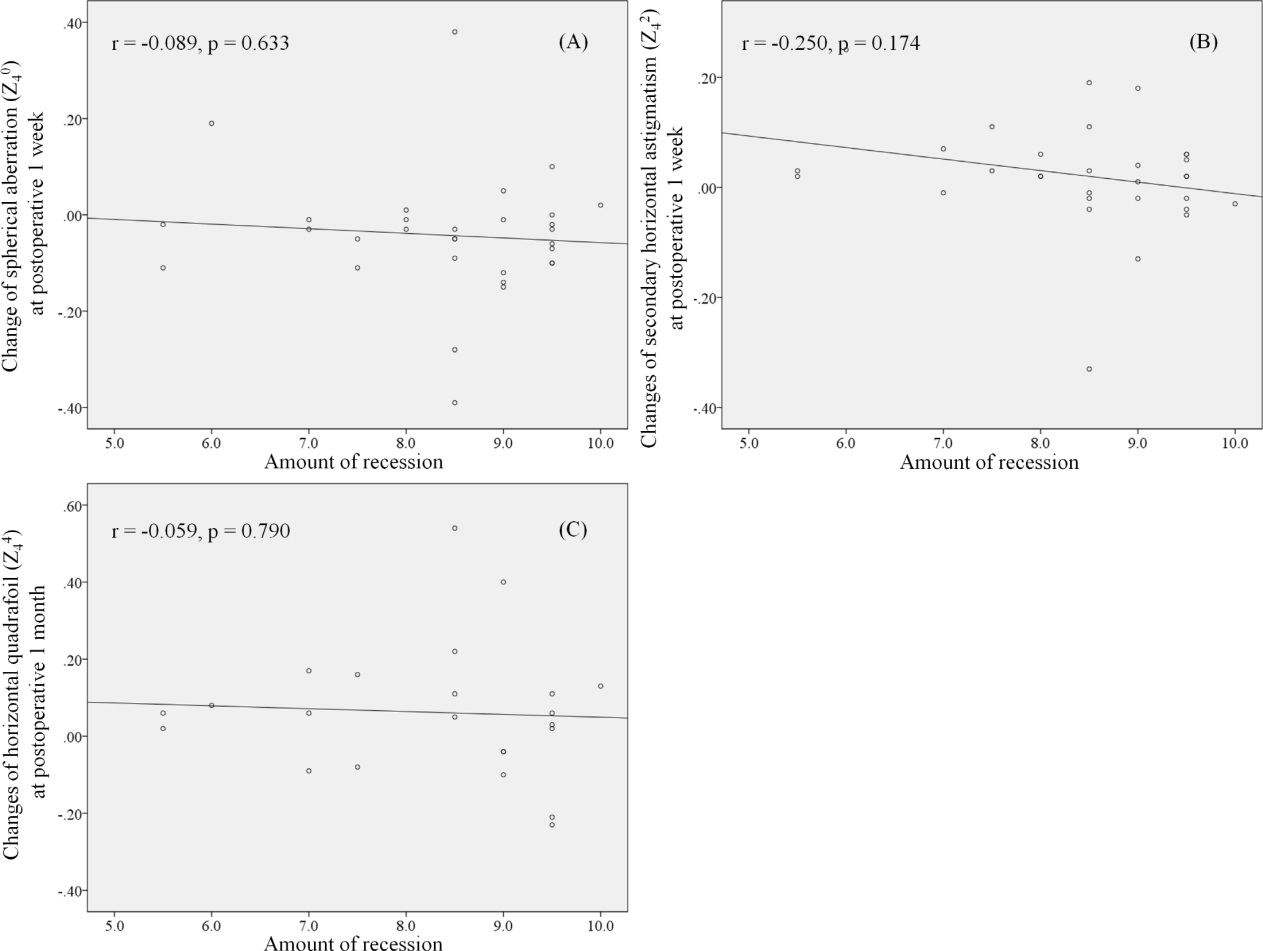
Correlation between recession amount and postoperative change of HOAs in group A (r = Pearson correlation coefficient). (A) Correlation between recession amount and change of spherical aberration (Z_4_^0^) at postoperative 1 week. (B) Correlation between recession amount and change of secondary horizontal astigmatism (Z_4_^2^) at postoperative 1 week. (C) Correlation between recession amount and change of horizontal quadrafoil (Z_4_^4^) at postoperative 1 month.

However, in the correlation analysis between the recession amount and the change of HOAs, with significant changes postoperatively in group A (spherical aberration (Z_4_^0^) and secondary horizontal astigmatism (Z_4_^2^) at 1 week, horizontal quadrafoil (Z_4_^4^) at 1 month), none of the values showed a significant association (Fig 1).

In group B, only vertical quadrafoil (Z_4_^-4^) showed a statistically significant change at postoperative 1 and 3 months (p = 0.023, 0.010, respectively). The changes of the other fourth-order aberration parameters did not attain statistical significance.

## Discussion

Several reports have noted changes in refraction caused by surgery on extraocular muscles [2, 3, 20–22]. Marshall reported that 60% of patients undergoing strabismus surgery showed a change in astigmatism [20]. Most changes of this kind have been thought to be related to changes in corneal curvature secondary to the reduction in the tension of the recessed extraocular muscle transmitted via the sclera to the cornea [4, 6–10]. A high incidence of increased with-the-rule astigmatism—up to 2 D of change—after horizontal muscle recession-resection procedures also has been reported [11].

Complete consideration of corneal topographic measurements, including analysis of the anterior, posterior, and total corneal curvature and HOAs, is important for better understanding of visual performance after strabismus surgery. One of the purposes of the current study was to evaluate and characterize the corneal structure by the Placido-dual Scheimpflug analyzer in patients who had undergone horizontal muscle surgery. In our study, there were significant changes in sim K, steep sim K, steep-Kpost, and TCP-steep K compared with the preoperative values. Sim K showed a significant change throughout the 3-month postoperative period. All of the steep K values showed a significant change at postoperative 1 month; however, they had reverted to the preoperative values by postoperative 3 months. We assumed that the mechanism of the temporal change of steep K is similar to that of the decreased tendency to regress to surgically induced astigmatism. Based on the above results, our study suggests that patients who have undergone ULR or BLR recession, relative to those who have undergone R&R, might need to change their glasses prescription later. In Group B, we did not find any topographic changes, not even at postoperative 1 week, indicating that none of the parameters of the corneal structure had, according to the Placido-dual Scheimpflug analyzer, changed after R&R. When the muscle was detached and placed behind its original insertion, the tension decreased. We considered the possibility that the resected muscle might compensate for the decreased tension of the recessed muscle, thus allowing patients to maintain a regular corneal topography after the R&R procedure.

The HOAs have recently been proven to affect vision quality [23, 24]. Important information on optical vision quality is provided by corneal wavefront profiles. Seo et al. reported that whereas the root mean square (RMS) of HOA was transiently increased after lateral rectus recession surgery, it returned to the baseline level after one month in children [25]. Among the individual Zernike coefficients, secondary astigmatism, quadrafoil, secondary coma, secondary trefoil, and pentafoil showed a tendency similar to that of the RMS of HOA [25]. Although we did not investigate more than fourth-order aberrations, the third-order aberrations were not changed significantly, whereas some of the fourth-order aberrations (spherical aberration (Z_4_^0^), secondary horizontal astigmatism (Z_4_^2^), horizontal quadrafoil (Z_4_^4^)) changed temporally after lateral rectus recession. As the presumed cause of increasing HOAs is considered to be decreasing tension on the sclera, we investigated the correlation between the recession amount relative to cornea proximity and postoperative change of HOAs in group A. However, the recession amount did not show significant correlations with any HOA values. Moreover, we found meaningful changes in the vertical quadrafoil (Z_4_^-4^) after R&R. On these bases, we can conclude that some variables of fourth-order aberrations changed after strabismus surgery, which might be one of the reasons why patients experience discomfort despite good visual acuity postoperatively.

There are some limitations to this study. First, as it was a retrospective study, evaluations of the repeatability and reproducibility of the examinations were not performed. Second, although the Placido ring images used by the device can be affected by tear-film irregularities, patients with dry eye were not excluded [26]. However, as a means of avoiding this effect, we made an effort to make patients blink completely immediately prior to each measurement. Third, we did not investigate data for the late postoperative period (more than 3 months). Because we analyzed data only until postoperative 3 months, we could not determine whether sim K would have maintained significant change after that or not. Further prospective clinical studies need to be conducted to clarify the long-term outcomes.

In conclusion, we showed that lateral rectus recession surgery induced partial changes in both corneal topographic measurements and HOAs. R&R induced change only in the vertical quadrafoil. Clinicians need to consider functional visual outcomes as they relate to surgically induced changes of corneal topographic measurements and HOAs.

## Supporting information

S1 File (XLSX)

## References

[1] Brum GS, Antunes-Foschini RM, abbud CM, Bicas HE. Variations of postoperative ocular alignment in patients submitted to strabismus surgery [article in Porttuguese]. Oftalmologia. 2011;74: 24–27. 10.1590/s0004-27492011000100006 21670903

[2] Dottan SA, Hoffman P, Oliver MD. Astigmatism after strabismus surgery. Ophthalmic Surg Las. 1988;19: 128–129. 3347457

[3] Fix A, Baker JD. Refractive changes following strabismus surgery. Am Orthopt J. 1985;35: 59–62.

[4] Nardi M, Rizzo S, Pellegrini G, Lepri A. Effects of strabismus surgery on corneal topography. J Pediatr Ophthalmol Strabismus. 1997;34: 244–246. 9253740 10.3928/0191-3913-19970701-13

[5] Preslan MW, Cioffi G, Min YI. Refractive error changes following strabismus surgery. J Pediatr Ophthalmol Strabismus. 1992;29: 300–304. 1432517 10.3928/0191-3913-19920901-09

[6] Killer HE, Bahler A. Significant immediate and long-term reduction of astigmatism after lateral rectus recession in divergent Duane’s syndrome. Ophthalmologica. 1999;213: 209–210. 10.1159/000027422 10202298

[7] Reynolds RD, Nelson LB, Greenwald M. Large refractive change after strabismus surgery. Am J Ophthalmol. 1991;111: 371–372. 2000909 10.1016/s0002-9394(14)72327-x

[8] Kwitko S, Feldon S, McDonnell PJ. Corneal topographic changes following strabismus surgery in Grave’s disease. Cornea. 1992;11: 36–40. 1559345 10.1097/00003226-199201000-00005

[9] Kwito S, Sawusch MR, McDonnell PJ, Gritz DC, Moreira H, Evensen D. Effect of extraocular muscle surgery on corneal topography. Arch Ophthalmol. 1991;109: 873–878. 2043078 10.1001/archopht.1991.01080060137042

[10] Rajavi Z, Mohammad Rabei H, Ramezani A, Heidari A, Daneshvar F. Refractive effect of the horizontal rectus muscle recession. Int Ophthalmol. 2008;28: 83–88. 10.1007/s10792-007-9116-z 17634864

[11] Thompson WE, Reinecke RD. The changes in refractive status following routine strabismus surgery. J Pediatr Ophthalmol Strabismus. 1980;17: 372–374. 7205517 10.3928/0191-3913-19801101-05

[12] Liu Z, Huang AJ, Pflugfelder SC. Evaluation of corneal thickness and topography in normal eyes using the Orbscan corneal topography system. Br J Ophthalmol. 1999;83: 774–778. 10381661 10.1136/bjo.83.7.774 PMC1723104

[13] Chen D, Lam AK. Intrasession and intersession repeatability of the Pentacam system on posterior corneal assessment in the normal human eye. J Cataract Refract Surg. 2007;33: 448–454. 10.1016/j.jcrs.2006.11.008 17321396

[14] Wang X, Dong J, Wu Q. Comparison of anterior corneal curvature measurements using a Galilei dual Scheimpflug analyzer and Topcon auto kerato-refractometer. J Ophthalmol. 2014:140628 Available from: http://www.ncbi.nlm.nih.gov/pmc/articles/PMC4055652/pdf/JOPH2014-140628.pdf 10.1155/2014/140628 24967095 PMC4055652

[15] Menassa N, Kaufmann C, Goggin M, Job OM, Bachmann LM, Thiel MA. Comparison and reproducibility of corneal thickness and curvature readings obtained by the Galilei and the Orbscan II analysis systems. J Cataract Refract Surg. 2008;34: 1742–1747. 10.1016/j.jcrs.2008.06.024 18812127

[16] Charman WN. Aberrations and myopia. Ophthalmic Physiol Opt 2005;25: 285–301 10.1111/j.1475-1313.2005.00297.x 15953113

[17] Lawless MA, Hodge C. Wavefront's role in corneal refractive surgery. Clin Exp Ophthalmol. 2005;33: 199–209. 10.1111/j.1442-9071.2005.00994.x 15807834

[18] Jahadi Hosseini SH, Abtahi SM, Khalili MR. Comparison of Higher Order Aberrations after Wavefront-guided LASIK and PRK: One Year Follow-Up Results. J Ophthalmic Vis Res. 2016;11: 350–357. 10.4103/2008-322X.194069 27994802 PMC5139545

[19] Al-Zeraid FM, Osuagwu UL. Induced Higher-order aberrations after Laser In Situ Keratomileusis (LASIK) Performed with Wavefront-Guided IntraLase Femtosecond Laser in moderate to high Astigmatism. BMC Ophthalmol. 2016;22: 16–29. 10.1186/s12886-016-0205-5 PMC4802649 27000109

[20] Marshall D. Changes in refraction following operation for strabismus. Arch Ophthalmol. 1936;15: 1020–1031.

[21] Denis D, Bardot J, Volot F, Saracco JB, Maumenee IH. Effects of strabismus surgery on refraction in children. Ophthalmologica. 1995;209: 136–140. 10.1159/000310599 7630620

[22] Hainsworth DP, Bierly JR, Schmeisser ET, Baker RS. Corneal topographic changes after extraocular muscle surgery. J AAPOS. 1999;3: 80–86. 10221799 10.1016/s1091-8531(99)70074-1

[23] Applegate RA, Howland HC. Refractive surgery, optical aberrations, and visual performance. J Refract Surg. 1997;13: 295–299. 9183761 10.3928/1081-597X-19970501-16

[24] Campbell CE. Improving visual function diagnostic metrics with the use of higher-order aberration information from the eye. J Refract Surg. 2004;20: S495–503. 15523965 10.3928/1081-597X-20040901-18

[25] Seo KY, Hong S, Song WK, Chung SA, Lee JB. Transient increase of higher-order aberrations after lateral rectus recession in children. Yonsei Med J. 2011;52: 527–529. 10.3349/ymj.2011.52.3.527 21488198 PMC3101054

[26] De Paiva CS, Lindsey JL, Pflugfelder SC. Assessing the severity of keratitis sicca with videokeratoscopic indices. Ophthalmology. 2003; 110: 1102–1109. 12799233 10.1016/s0161-6420(03)00245-8

